# Supplementary material for: Cardiometabolic risk factors in social housing residents: A multi-site cross-sectional survey in older adults from Ontario, Canada
Source: PLoS One. 2024 Apr 4;19(4):e0301548. doi: 10.1371/journal.pone.0301548 (PMC10994361; doi:10.1371/journal.pone.0301548)
Supplement: Attachment — Submitted filename: Response to Reviewers.docx [file pone.0301548.s002.docx]

February 22^nd^, 2024

Dear Mr., Nakhostin-Ansari

**Re: Manuscript ID PLOS ONE Decision: Revision required [PONE-D-23-35505] - [EMID:bf543bfab3c6e8d5] entitled “Cardiometabolic risk factors in social housing residents: a multi-site cross-sectional survey in older adults from Ontario, Canada”**

Thank you very much for the timely and highly constructive suggestions from your reviewers that have greatly improved the clarity of the study. Below is a detailed response and actions for all of the recommendations. We thank you and the PLOS reviewers for assisting us in developing this important study.

**Reviewers’ Comments**

| **#** | **Reviewer / editorial comment** | **Correction/clarification** |
| --- | --- | --- |
|  | **Reviewer #1** |  |
| 1 | Technical Soundness: The manuscript demonstrates a high degree of technical soundness. The research methodology appears well planned and executed. The experimental design, data collection, and analysis are all appropriately documented. The study's objectives are clear, and the research questions are logically addressed. The use of appropriate methods and techniques in data collection and analysis enhances the manuscript's credibility. | Thank you for this feedback. |
| 2 | Data and Conclusions: The data presented in the manuscript are comprehensive and well organized. It is evident that the data supports the conclusions drawn. The results align with the research objectives, and the conclusions are well founded. The logical flow of the manuscript aids in understanding the significance of the findings. This alignment between the data and the conclusions significantly strengthens the manuscript's overall quality. | Thank you for this feedback. |
| 3 | Statistical Analysis: The statistical analysis in the manuscript is both appropriate and rigorous. The methods utilized for data analysis are well described, and the statistical tests chosen are relevant to the research questions. Moreover, the presentation of results in tables, figures, and the accompanying discussion contributes to the overall clarity of the statistical analysis. | Thank you for this feedback. |
| 4 | Presentation and Clarity: The manuscript is well structured and presented in an intelligible fashion. The logical flow from introduction to methods, results, and discussion aids readers in following the research narrative. The tables are appropriately placed and add value to the text, helping to visualize the data and results. | Thank you for this feedback. |
| 5 | In conclusion, manuscript is of high quality, both in terms of its technical content and presentation. The study is methodologically sound, the data analysis is robust, and the conclusions are well supported by the data. | Thank you for this feedback. |
| 6 | Recommendations to the authors: Health related issues have been considered, however I recognized some missing information related to access of social housing residents to primary health care and medicines required to manage chronic conditions, including access to medicines used in diabetes, hypertension, etc. Such data will provide additional strong evidence to policy decision makers in development of a specific program to address needs of social housing residents in health care | We have added the following context to the introduction, and it is already discussed in the discussion in line 247/248, however, we do not have the ability to add new data into this paper about these considerations:  Though this population has been shown to have health care providers ^1^, there are some challenges with accessing the healthcare in a timely fashion ^2^. |
|  | **Reviewer #2** |  |
| 1 | Descriptive study of a local vulnerable population of social housing inhabitants in Ontaria Canada, whose generalizability and public health implications are lacking. | - We have added content to the discussion now to address these comments. - There are implication for public health which we have clarified. - There is a line that does comment on the generalizability already in the discussion page 13. . - This is an implication for public health page 15. |
| 2 | Line 148: How did you ascertain that BMI was expected to be missing not at random? | - A better description has been placed in line 146. - This section has been substantially revised to address the reviewer’s important observations and criticisms, and to improve its clarity. |
| 3 | Definition of CVD used in the current study should be clarified. | The definition of CVD has been added in the Objectives section. |
| 4 | The analysis plan of objectives 2 – 4 in lines 91 – 93 is lacking. | - We have added the appropriate analysis now in the statistical analysis section. - A better description has been placed in line 146. |
|  | **Reviewer #3** |  |
| 1 | Although the paper's significance is understandable, the introduction can be paraphrased due to unnecessary repetition and needs better structure. Several times, you mentioned the modifiable and behavioral risk factors. Also, you repeatedly say the General purpose of the study ( lines 66-67, 71-72, and 95). | We have modified the introduction and other parts of the paper in tracked changes to demonstrate where we have cut the redundant phrasing – thank you for the suggestion. |
| 2 | In Table 1, please add a percentage (%) mark in the related parenthesis to ease reading. Also, add a definition of the abbreviation in the footer of the tables ( CVD,…) | - Percentage (%) was added. - Definition of the abbreviation in the footnote was provided. |
| 3 | It would be better to describe the ranges (definition) of variables you asked for from participants in the method section. For example, the readers can only understand if they read the questionnaires separately) what low or high drinkers mean precisely. | - Low & high drinkers have now been specified as footnote in table 4. |
| 4 | 4. Reports of health-related quality of life in the total study population( unless the cardiometabolic disease) can be informative, too. Add the entire population column in Table 5. | This table associates QoL with CVD and Diabetes specifically and although interesting we respectfully disagree that it makes sense to comment on the total study population. |
| 5 | The descriptive reports of this article are important and support the purpose of the study well. On the other hand, analytical reports(chi-squares) often need to convey something meaningful to the reader, considering the purpose of the study. In fact, the purpose of the study, as evident in the discussion section by comparing the descriptive reports with the society by the authors, is that the reports of P values do not add anything new to the reader. The absence of most of them and only the descriptive report is enough for the study. In this study, we do not want to compare and correlate the effect of risk factors in diabetic or cardiac patients, but only their distribution in the targeted population is essential. | Thanks for this comment. We agree that we were trying to describe our population with others in Canadian society to place the context of this vulnerable population firmly in the minds of health services decision makers. |
| 6 | 6. PLOS authors have the option to publish the peer review history of their article. If published, this will include your full peer review and any attached files. | Yes, we agree to this. |

1.	Agarwal G, Angeles R, Pirrie M, McLeod B, Marzanek F, Parascandalo J, Thabane L. Evaluation of a community paramedicine health promotion and lifestyle risk assessment program for older adults who live in social housing: a cluster randomized trial. CMAJ. 2018;190(21):E638-E47

2.	Pirrie M, Agarwal G. Older adults living in social housing in Canada: the next COVID-19 hotspot? Canadian journal of public health = Revue canadienne de sante publique. 2021;112(1):4-7.
