# Supplementary material for: Cardiometabolic risk factors in social housing residents: A multi-site cross-sectional survey in older adults from Ontario, Canada
Source: PLoS One. 2024 Apr 4;19(4):e0301548. doi: 10.1371/journal.pone.0301548 (PMC10994361; doi:10.1371/journal.pone.0301548)
Supplement: Attachment — Submitted filename: Plos response to reviewers.docx [file pone.0301548.s003.docx]

| **Comments** | **Response** |
| --- | --- |
| 1. If the authors have adequately addressed your comments raised in a previous round of review and you feel that this manuscript is now acceptable for publication, you may indicate that here to bypass the “Comments to the Author” section, enter your conflict of interest statement in the “Confidential to Editor” section, and submit your "Accept" recommendation.  Reviewer #2: All comments have been addressed  Reviewer #3: All comments have been addressed | Thank you for agreeing that the requirements have been met. |
| 2. Is the manuscript technically sound, and do the data support the conclusions?  The manuscript must describe a technically sound piece of scientific research with data that supports the conclusions. Experiments must have been conducted rigorously, with appropriate controls, replication, and sample sizes. The conclusions must be drawn appropriately based on the data presented.  Reviewer #2: Partly  Reviewer #3: Yes | We believe based on the required comments that we have met the requirements requested of this point. |
| 3. Has the statistical analysis been performed appropriately and rigorously?  Reviewer #2: Yes  Reviewer #3: No | We are aware that the 2 reviewers have differing opinions as to the statistical analysis that we performed, however we believe that this is common practice in research for there to be disagreement amongst academics over personal preference for styles of analysis performed. We are confident in our analytical techniques that we used, as several members of the research team have had extensive and rigorous statistical training at a doctorate level. Furthermore reviewer #3 commented in the previous review that inferential analysis was not required and that descriptive analysis was enough. Other reviewers might want to see the inferential analysis as well so we believe presenting both in this paper is the better option. |
| 4. Have the authors made all data underlying the findings in their manuscript fully available?  The [PLOS Data policy](http://www.plosone.org/static/policies.action#sharing) requires authors to make all data underlying the findings described in their manuscript fully available without restriction, with rare exception (please refer to the Data Availability Statement in the manuscript PDF file). The data should be provided as part of the manuscript or its supporting information, or deposited to a public repository. For example, in addition to summary statistics, the data points behind means, medians and variance measures should be available. If there are restrictions on publicly sharing data—e.g. participant privacy or use of data from a third party—those must be specified.  Reviewer #2: Yes  Reviewer #3: Yes | Thank you for agreeing that the requirements have been met. |
| 5. Is the manuscript presented in an intelligible fashion and written in standard English?  PLOS ONE does not copyedit accepted manuscripts, so the language in submitted articles must be clear, correct, and unambiguous. Any typographical or grammatical errors should be corrected at revision, so please note any specific errors here.  Reviewer #2: Yes  Reviewer #3: Yes | Thank you for agreeing that the requirements have been met. |
| 6. Review Comments to the Author  Please use the space provided to explain your answers to the questions above. You may also include additional comments for the author, including concerns about dual publication, research ethics, or publication ethics. (Please upload your review as an attachment if it exceeds 20,000 characters)  Reviewer #2: All concerns were addressed by investigators and I have no new queries. Therefore, no further comments.  Reviewer #3: Thanks to the authors for the interesting article and valuable revisions. The authors have made the necessary corrections precisely. However, as I mentioned in a comment, Reports of health-related quality of life in the total study population, unless the cardiometabolic disease, can be informative too. Although the authors wanted to compare the two major Cardiometabolic disorders groups, the reports of the total population as a vulnerable group can be informative, too. There is no further comments | Thank you for agreeing that the requirements have been met.  Thank you reviewer #3 for pointing out that QoL can be informative - we were restricted by the space requirements of the tables and have therefore decided not to add another extra column in this case, which would make the table very cramped and hope you can accept this decision. We are preparing a separate paper that looks at QoL specifically in a larger social housing population in much more detail and do not feel it is relevant to place it here. |
| General Comment:  Please review your reference list to ensure that it is complete and correct. If you have cited papers that have been retracted, please include the rationale for doing so in the manuscript text, or remove these references and replace them with relevant current references. Any changes to the reference list should be mentioned in the rebuttal letter that accompanies your revised manuscript. If you need to cite a retracted article, indicate the article’s retracted status in the References list and also include a citation and full reference for the retraction notice. | Thank you for this - we have now checked the manuscript fully and made these 4 changes:  **reference #3 substituted:**  3. Canada PHAo. Center for Surveillance and Applied Research; 2017. substituted to  3. The 2017 Canadian chronic disease indicators SC. Health Promotion and Chronic Disease Prevention in Canada: Research, Policy and Practice. 2017;37(8):248.  **reference #5 with minor changes updated:**  5. Association ON-PH. Aging in place in social housing. 2016. Changed to  5. Ontario Non-Profit Housing Association (ONPHA). Aging in place in social housing Prepared by ONPHA 2016.  **reference #15 with minor change updated:**  15. Canada. S. Canadian Community Health Survey - Healthy Aging (CCHS) 2009. Changed to  15. Statistics Canada. Canadian Community Health Survey - Healthy Aging (CCHS) 2009.  **reference #38 with minor change updated:**  38. Canada. S. Highest level of education by geography:. Canada, provinces and territories. Changed to  38. Statistics Canada.Highest level of education by geography:. Canada, provinces and territories. |
